# Becoming fathers, becoming caregivers: A qualitative exploration of intersectional influences shaping caregiving in an urban poor South Indian setting

**DOI:** 10.1371/journal.pone.0334717

**Published:** 2025-10-23

**Authors:** Eunice Lobo, Joshua Jeong, Giridhara Rathnaiah Babu, Debarati Mukherjee, Onno C. P. van Schayck, Prashanth Nuggehalli Srinivas

**Affiliations:** 1 Indian Institute of Public Health - Bengaluru, Public Health Foundation of India, Bengaluru, India; 2 PHFI Centre for Developmental and Lifecourse Research, Bengaluru, India; 3 Centre for Health Systems, Institute of Public Health Bengaluru, Bengaluru, India; 4 Department of Family Medicine, Care and Public Health Research Institute (CAPHRI), Maastricht University, Maastricht, The Netherlands; 5 Hubert Department of Global Health, Rollins School of Public Health, Emory University, Atlanta, United States of America; 6 Department of Population Medicine, College of Medicine, QU Health, Qatar University, Doha, Qatar; Rural Women's Social Education Centre, INDIA

## Abstract

**Background:**

Caregiver engagement is crucial for early child development; however, research on paternal involvement remains limited, particularly in urban settings of the Global South. This exploratory study aimed to understand how fathers’ lived experiences and aspirations, along with systemic inequities, shape their parenting practices in urban poor settings in Bangalore, South India.

**Methods:**

Ten fathers of children aged 4–6 years from low socio-economic backgrounds in the MAASTHI birth cohort were purposively selected for in-depth interviews, conducted using a pre-tested topic guide in *Hindi* and *Kannada*. All interviews were transcribed, translated, and analysed using a thematic analysis approach.

**Results:**

Fathers prioritized their children’s education and safety, often viewing financial provision as their primary role due to ingrained gender norms and economic hardship. Most worked long hours in informal employment, thereby limiting their participation in daily caregiving, which was typically handled by their mothers. Fathers’ own childhood experiences influenced their parenting, with those who experienced adversity often aiming to break intergenerational cycles by being more emotionally present and supportive. While structural barriers limited involvement, many fathers expressed a strong desire to be more engaged, thereby challenging traditional roles. Safety concerns in their neighbourhoods further shaped protective parenting practices. Despite these constraints, some fathers reported that they preferred spending time with their families and participated in co-parenting through shared decision-making and engaging in play.

**Conclusion:**

This study highlights the intersectionality between gender, socio-economic status, and intergenerational adversity in shaping fathering practices. To promote inclusive caregiving, early childhood programmes must actively include fathers and address both individual and structural barriers that constrain their involvement.

## Background

Most existing research on early childhood development (ECD) focuses on maternal engagement, showing strong links to child cognitive, emotional, and social growth [1,2]. However, paternal involvement remains significantly underexplored, particularly in marginalized urban poor settings in the Global South [3]. This gap is concerning, given the growing evidence that fathers play a unique and complementary role in shaping children’s development, influencing cognitive and socio-emotional regulation, as well as long-term educational outcomes [4–6]. While global frameworks like the Nurturing Care Framework [7] highlight the importance of engaging multiple caregivers, including a thematic brief that spotlights the importance of fathers [8], in practice, male caregivers remain overlooked in ECD policies and interventions, reinforcing the notion that child-rearing is primarily a maternal responsibility. At the same time, efforts that intentionally aim to engage fathers are also not necessarily easy or seamless due to the entrenched gender norms and unequal caregiving expectations, with many fathers primarily focusing on provider roles, which may partly explain their limited representation in caregiving research and thus this research gap.

Nevertheless, emerging evidence from the Global North shows that greater paternal involvement in childcare is linked to a wide range of positive outcomes, including improved marital satisfaction [9], fatherhood confidence [10,11], and children’s well-being [12], while also promoting gender equality and work-life balance [13]. Recognizing and supporting the factors that encourage men’s caregiving can strengthen families and foster more equitable parenting in diverse cultural contexts. However, research from the Global South remains sparse where paternal caregiving has been characterized singularly in terms of economic provisions, without also considering men’s nurturing care for early childhood development [14].

In urban poor communities of South Asia, including Bangalore, fathers often navigate unstable employment, rigid gender norms, and limited or no access to parental support programmes [15]. Many men work long hours in the informal sector without job security or paid leave, making involvement or even participation in caregiving difficult [16]. Simultaneously, deeply ingrained social norms around masculinity position fathers primarily as financial providers rather than as interactively engaged in nurturing care, as noted in settings in the Global North [17,18]. This exclusion not only limits opportunities for engagement but also reinforces gendered divisions of care. Despite these constraints, many fathers demonstrate aspirational caregiving, trying hard to engage in ways that challenge traditional norms [19]. However, most research and policy discourses predominantly frame fathers through the lens of absenteeism or a lack of interest in caregiving [20].

Beyond coexisting factors such as men’s individual attitudes towards caregiving, poverty, and social norms, intergenerational experiences also shape how fathers approach caregiving. Using Bandura’s Social Learning Theory framework [21], studies have noted that fathers may model their own childhood experiences, either replicating or consciously rejecting past caregiving practices. Those who experienced neglect or adverse childhood experiences may not participate in caregiving due to a lack of confidence in their parenting abilities, while others may engage in compensatory parenting, intentionally providing a more nurturing environment than they received [22,23]. These complexities highlight that paternal engagement follows a lifecourse approach – thus it is not solely determined by factors during adulthood but also influenced by men’s childhood experiences, and evolving aspirations. This is supported by longitudinal research showing that early paternal involvement can predict later caregiving, even when adjusted for structural factors such as employment hours [24]. Similarly, Izenstark & Middaugh found that early family-based activities, such as outdoor experiences, can shape adult preferences and parenting behaviours, illustrating how early exposures contribute to intergenerational patterns of engagement [25].

Although research on fatherhood in the Global North has expanded, much of it is centered on dual-income households or middle-class families, leaving a critical gap in understanding how fathers in the Global South, particularly in urban areas, navigate caregiving. To address these gaps, we conducted a qualitative study to explore paternal caregiving in urban poor settings of Bangalore, where gender norms are relatively rigid. Our objectives were to: i) explore how contextual factors shape low‑income fathers’ caregiving practices in urban poor neighbourhoods of Bangalore; ii) characterize the caregiving styles and activities of fathers in caregiving of their children. This study draws on an intersectionality lens to examine how caregiving among low-income fathers in urban South India is shaped by the interaction of multiple axes of identity and structural inequities, including socio-economic status, gender norms, work precarity, and intergenerational differences. Hence, we explore how the combination and intersection of factors shape fathers’ roles, aspirations, and practices. While recent qualitative studies in India have examined paternal roles in child care and adolescent health [26–28], this study uniquely highlight fathers’ caregiving experiences, aspirations, and styles and offer a deeper, context-specific understanding of fatherhood in low-resource settings. These understandings are essential for shaping inclusive policies and interventions that acknowledge fathers’ vital role in child well-being, ensuring childhood development frameworks are more reflective of diverse caregiving realities.

## Materials and methods

### Study design and participants

The urban metropolis of Bangalore, the capital city of Karnataka in southern India, has witnessed rapid urban expansion in recent decades. With around one-third of its residents living in slums or informal settlements among the population of nearly 14 million, families often face limited access to housing, services, and economic opportunities [29]. As part of the Nutritional, Psychosocial, and Environmental Determinants of Neurodevelopment and Child Mental Health (COINCIDE) study, we conducted qualitative research to explore caregiving practices in urban settings of Bangalore. The COINCIDE was nested within the Maternal Antecedents of Adiposity and Studying the Transgenerational Role of Hyperglycemia and Insulin (MAASTHI) cohort. The MAASTHI birth cohort is based in urban Bangalore, established in 2016 with recruitment until 2019 and annual follow-up of mother-child dyads. Details of the cohort, and the COINCIDE study are published elsewhere [30–32].

For this exploratory study, we approached fathers in this cohort to participate in the in-depth interviews. This was the first time fathers were recruited for participation since mothers were recruited from 2016 to 2019. Over the years, multiple socio-economic indicators including household income, assets, education, and occupation have been collected to characterise these neighbourhoods as low-income settings. We further purposively selected fathers to ensure variation in age, occupation, education, and family structure based on the information collected during the quantitative sweep of the COINCIDE study. Hence, a total of 10 fathers were recruited from 1 April to 30 June 2024 to capture the diverse caregiving experiences of fathers, particularly those with children aged 4–6 years. Participants provided written informed consent if they were willing to engage in an in-depth interview regarding their caregiving roles and experiences. Fathers who had a limited ability to participate due to language or communication barriers were excluded from the study. Among the 12 fathers approached, two declined to participate, citing work obligations that prevented their availability for interviews during evenings or weekends. This study adheres to the Consolidated Criteria for Reporting Qualitative Research (COREQ) guidelines [33] (S1 File).

### Tool development, training, data collection, and quality assurance

For our exploratory study, ten face-to-face in-depth interviews (IDIs) were conducted between April and June 2024 to explore the fathers’ experiences with caregiving, challenges, and factors influencing their caregiving role, using a semi-structured topic guide. The topic guide, along with the information sheet and consent form, was developed by the first author (EL) with inputs from the senior author (PNS) and refined after pre-testing prior to interviews. Based on the participants’ preference, the in-depth interviews were conducted in either *Hindi or Kannada* languages as per the fathers (participants) preference. Interviews were scheduled at times convenient for the fathers to minimize disruption to their daily routines and work, including late evenings and Sundays. All interviews were conducted at the participants’ homes to ensure comfort and privacy.

Prior to the in-depth interviews, written informed consent was obtained from all participants after explaining the study objective. The first author (EL) conducted interviews in *Hindi*, while a trained female interviewer, fluent in *Kannada*, conducted interviews in *Kannada*. This approach allowed for broader representation of fathers from *Hindi*-speaking and *Kannada*-speaking households. The assessor with a master’s in public health degree, was already well-versed with the study due to her association as a quantitative assessor for the COINCIDE study. She was then trained in qualitative research by the first author and additionally supervised during the interviews. The interviews’ duration was between 25 and 45 minutes, and were audio recorded after consent. Following completion, all audio recordings were de-identified and uploaded to a secured drive accessible only to the senior team members. Interviews were then transcribed and translated verbatim into English by outsourced professional translators with experience in qualitative research projects. To ensure the accuracy of the translations, a quality check was performed by both interviewers, who reviewed the translated transcripts against the original recordings. This step was critical to maintain the integrity of the participants’ responses and minimize potential translation errors.

Given the exploratory focus of this study and the substantial resource constraints faced, the authors employed a purposive sample of ten face-to-face in‑depth interviews. Using Braun and Clarke’s guidelines for thematic saturation [34], coding was carried out iteratively, with codes reviewed after each set of interviews, and it was observed that by Interview 10, the collected information was sufficient to address the primary objective of this exploratory study, with little to no new information emerging. A coding matrix (see S2 File) mapping codes and themes across all interviews further demonstrates that all major themes and sub-themes were consistently represented. Hence, in consultation with senior co-authors, and mindful of limited resources, data collection was stopped after 10 interviews.

### Data analysis

The transcribed and translated transcripts were analysed using thematic analysis [35]. The first author familiarized herself with the data through repeated reading of the transcripts. She developed an initial version of the codebook informed by both the study objective and the types of responses that emerged during the familiarization process. Next, the first author proceeded with the formal coding phase using NVivo 12 Plus [36]. This allowed for systematic organization and traceability of codes. She applied the codes to the transcripts, continually refining the codebook as new themes and patterns emerged. This process involved collating codes, reviewing patterns with senior authors, and defining and naming themes. To ensure reliability and reduce bias, coding decisions and emerging themes were regularly reviewed and discussed with senior authors. An intersectional lens also guided the analytic process, based on father’s narratives [37]. Hence, patterns of code co-occurrence were discussed among the team and used to inform interpretation. These steps collectively ensured that the thematic analysis was rigorous, credible, and accurately reflected the lived experiences and practices of fathers in the study.

### Reflexivity

EL, an Indian woman pursuing a PhD in public health, trained in qualitative research, and a mother to a young child, reflected on how her gender and parental role influenced her interactions with fathers and shaped her understanding of the data. Keeping in mind her own experiences as a mother, she was mindful of how these interactions could both facilitate rapport and potentially influence the narratives shared. The senior author, PNS, an expert in qualitative research and health inequities, contributed expertise in study design and methodology. His role as a father also gave an insider perspective, aiding in the analysis. His experience spanning urban, tribal, and rural communities particularly in health inequities brings a deeply personal and insightful perspective, grounded in his role as a father. JJ, a global specialist in early childhood development and father involvement, offered critical feedback and shared insights from other Global South settings to improve the analysis while relying on the team’s local expertise to ensure the analysis remained culturally grounded. GRB, DM, and OvS, all parents, engaged in reflective discussions to explore how their caregiving experiences informed the theme development. They recognized that their personal caregiving roles could influence their interpretations and made efforts to differentiate between personal biases and participants’ experiences and practices. All but two authors have previously worked, and continue to work, in urban poor settings in India. Many have spent several years engaging in research and community-based work in these areas, which has contributed to a deeper understanding of the local cultural context and caregiving practices. However, they also acknowledged that their middle-class backgrounds might lead to assumptions about participants’ experiences, thus requiring continuous reflexivity. Therefore, during the several rounds of discussions regarding the coding and emerging themes, authors consciously kept in mind their own potential biases and tried to avoid their influence on the interpretation of participants views and practices, so that the analysis remained grounded in participants’ realities.

### Ethical considerations

Our study was approved by the Institutional Ethics Committee of the Indian Institute of Public Health-Bengaluru (*vide* IIPHHB/TRCIEC/214/2021) on 20 May 2021, with annual approval until end of the study. Participants were given detailed information about the study through an information sheet, and written informed consent was obtained before the interview. Fathers were gifted a stainless-steel plate, costing INR 100 (~USD 1.2), as a token of appreciation.

## Findings

Table 1 shows the socio-demographic profile of the ten fathers interviewed for this study. The fathers were aged between 28 and 42 years, with varying educational backgrounds ranging from third grade to high school as their highest educational attainment. Most were self-employed or engaged in informal occupations such as driving, demolition work, and sales. Household sizes varied significantly, ranging from four to fourteen members, with self-reported, approximate monthly incomes of INR 10,000–1,00,000 (approximately USD 117–1,170).

**Table 1:** Characteristics of fathers interviewed via in-depth interviews (n = 10).

Caregiver ID	Age (years)	Child age (years)	Child sex	Education	Occupation	Household members	Approx. monthly HH income (INR)
Father_1	34	6	Male	Primary school	Self-employed (Car accessories)	4	25,000
Father_2	40	5	Male	High school	Self-employed (Transport)	5	40,000
Father_3	36	5	Female	Primary school	Self-employed (Demolition)	6	1,00,000
Father_4	31	4	Male	Primary school	Labour work	4	10,000
Father_5	38	6	Male	Primary school	Auto driver	4	15,000
Father_6	28	5	Female	High school	Driver	6	20,000
Father_7	42	4	Male	High school	Self-employed (Electrician)	4	15,000
Father_8	31	4	Female	High school	Salesperson	4	25,000
Father_9	34	4	Male	High school	Driver	14	30,000
Father_10	36	5	Male	High school	Mechanic	9	10,000

INR: Indian Rupee; 1 USD = 85.56 INR as on July 2025

This study examines caregiving among fathers in low-income urban communities using an intersectional lens. We explored how fathers’ experiences are influenced by the combined effects of childhood experiences, insecure work, limited education, gender norms, social expectations, and other factors. The findings are organized under three overarching themes: (A) individual factors, (B) systemic factors, and (C) parenting styles based on their lived experiences.

Each theme is supported with illustrative quotes that highlight their voices.

A. **Individual factors**
  1. **Childhood experiences as a catalyst for parenting**

Fathers’ reflections on their adverse childhood experiences profoundly shaped their parenting philosophies. Fathers described growing up in environments of poverty, neglect, or familial instability, and consequently, these experiences motivated them to break cycles of adversity for their children actively. While their personal motivations were strong, they were also influenced by their economic situation and the way they had been raised.

### Own childhood experiences.

Fathers frequently reflected on their own childhoods, recognizing how their adverse experiences shaped their present-day parenting. Many sought to shield their children from similar hardships.

*“Earlier even during school holidays, my father was concerned that we might waste time at home, so he put us to work. You could say I didn’t continue my schooling. We also had financial difficulties at the time, and we didn’t even have a proper home. Due to these problems, I continued working and didn’t return to school. I’ve been working ever since.”* (Father_1)

*“Our parents have not spent time with us, as they did not have the time. I don’t want the things to be repeated now. I want to be his friend. He does not have a brother; both are his sisters. So, I want to make him comfortable with me.”* (Father_2)

*“Because our future has gone in vain. So I want to save their future. That is why I take interest in their whereabouts, take them out when I can, spend time with them at home.”* (Father_4)

### Breaking cycles.

Many fathers experienced neglect or hardship in their own childhoods, often linked to structural poverty or rigid parenting norms. Fathers expressed a commitment to ensuring their children’s futures were free from the hardships they had faced. They viewed it as their responsibility to provide not only financial security but also a nurturing environment at home. They believed this was important to break cycles of disadvantage, ensuring their children had opportunities they never had.

*“The love my father showed me, I feel I am giving my son even more than that. I may not always have enough time for him, but we make sure to manage. Earlier times were different, so our parents were taking care of us in a different way. Nowadays, we are taking care of our children differently.”* (Father_10)

*“I am trying my best to give them a bright future. I want to be known by my children’s name. I protect them from all the wrong things. It is my desire to teach them a lot. I will do my best to give them the best education. I will do whatever it takes to provide them with a good education, so that they can have a good life ahead. I will work harder, but their life should be good.”* (Father_4)

*“We do not allow our children to play outside. They can easily pick up bad habits from others. That is why I give them more time, and I try to spend more time with them. There will not be any benefit of earning if they pick up these bad habits. Our children are our assets, I am trying to give them what I did not get, we have struggled a lot from childhood, I do not want my children to struggle.”* (Father_3)

2. **Aspirations as a driver of involvement**

Fathers’ caregiving practices were deeply influenced by their aspirations for their children’s futures. These aspirations, often motivated active involvement in their children’s life.

### Education as a pathway to success.

Education was unanimously prioritized as a pathway to success. They viewed education as a crucial pathway to success – an opportunity they themselves had lacked. They believed that education can help children avoid hardships and build a better future. Fathers desired to equip their children with the knowledge and skills needed to become independent and successful in life.

*“Earlier, the environment was not as good. I am trying to provide a better one now. I am more focused towards education and values, we did not have the opportunity to study because of our circumstances or our parents did not have time for us, but I want to give my children both good values and good education.”* (Father_3)

*“My only priority is my children’s future. Since I am not educated, I faced many challenges, but my situation was different back then. Now that we have a home and enough food to eat, we are more focused on our children’s education and their future. If we give them a good upbringing, they will have a better future.”* (Father_1)

*“It is our responsibility as parents to look after their future. Right now, they are children, so we have to decide for them. When they become mature, they can decide. We should try to make them understand the importance of studying and building their future. We work hard to help them stand on their own feet someday.”* (Father_5)

### Children as a source of joy and purpose.

Fathers derived significant emotional fulfilment from their children’s achievements, viewing parenting with a sense of purpose. Witnessing their children’s curiosity, kindness, or even academic success reinforced their involvement in caregiving. They also took pride in their children’s talents and aspirations, supporting their interests and envisioning bright futures for them.

*“If my daughter is happy, then we will be happy. We may have more tension outside, but if our children are happy, then we will be happy. These things bring us more happiness.”* (Father_6)

*“I try to get my son whatever he needs. I enjoy buying my children new toys or some new clothes. They feel very happy when they receive them. I also feel very happy to see them like that.”* (Father_9)

*“He plays cricket so well. He is actually very good at cricket. Seeing this, I ordered a leather ball for him, and he was not frightened to play with it. He plays with that ball also. I always have this thought in my mind to make him a player someday in the future. So, let us see (smiles).”* (Father_2)

B. **Systemic factors**
  1. **Tradition and transition**

Fathers’ involvement was shaped by systemic gender norms and changing generational expectations. While many played the “provider” role due to work demands and social norms, others sought more engaged parenting, informed by reflections on their own childhood experiences and differences across generations.

### Restrictive gender norms.

Most fathers assumed the provider role, prioritizing financial stability over hands-on caregiving. This was particularly pronounced in low-income households, where fathers worked long hours. On the other hand, mothers were seen as the primary caregivers, responsible for managing the home and overseeing children’s daily lives. Many referenced gender norms to explain these traditional gender roles and divisions in maternal versus paternal caregiving.

*“Every time children will be with the mother, so she is responsible for their upbringing. I feel that since the father is at work, he can provide finances but care she needs to take.”* (Father_9)

*“I believe it is the mother’s responsibility to take care of the children and raise them, because I go to work in the morning and am not home all day. In the evening, I get home, freshen up, eat, and then go to sleep, so I do not have much time. That is why it is the mother’s responsibility. She is the one watching over them, seeing where they are going and coming from. It is all on the mother. Our responsibility as fathers is to earn.”* (Father_6)

*“I work hard and I bring money home. My wife carefully works at home and looks after my children and home as I cannot handle everything. We both need to manage.”* (Father_10)

### Generational differences.

Fathers reflected on their own upbringing and the differences in their parenting styles compared to those of their parents. They acknowledged that times were different and changing and recognized the need to adapt to the times. They were determined not to follow the same parenting style that was often distant, neglectful, or even harsh. They wanted to provide a warm and supportive environment for their children.

*“Back then, we used to be scared of our parents and always stayed behind them quietly. They were strict with us, and because of that strictness, we ate whatever they gave us and studied well. But today’s 4G generation doesn’t understand the language of strictness. They are happier and more willing to listen when you talk to them politely, or when you are fulfilling their demands, even if small.”* (Father_2)

*“In earlier days, they used to beat us for small reasons, but now it is different. We would like to be friendly with them. I have not disciplined my children as my parents did. Nowadays, we cannot be the same; we also have to change with time.”* (Father_6)

*“My parents were careless, but we take care of our children. We are more careful about their education, where they play, what they eat.”* (Father_8)

2. **Socio-economic hardships and societal pressures**

Fathers faced socio-economic hardships, and time constraints that restricted their time to provide care. Additionally, community safety concerns compelled them to adopt protective parenting strategies, restricting outdoor play and social interaction. Further societal pressures through expectations also shaped fathers’ caregiving practices. Together, these factors emphasize the systemic challenges that directly impact well-being and child development.

### Economic and time constraints.

Fathers expressed the challenges of balancing work and family time due to job-related constraints. With irregular or demanding work schedules, they often struggle to spend quality time with their children, as missing even a single workday affects their income. Some fathers desired more involvement in the lives of their children. They were bound by the amount of time, but they tried to engage with them and show their care through weekend outings or games to compensate for their absence.

*“Yes, I want to spend more time with both my children, but my work is not fixed. If I do not go to work one day, I will make no money. So it is difficult to manage time with them.”* (Father_5)

*“I will be on duty even on Sundays, so I am not able to take my children anywhere. Mostly, they (children) spend time with my brother’s children or playing outside. Once in a while, I take them to the park to play. We go only if I don’t have a duty. But he (son) enjoys it a lot when we go there. It is nearby, so we can go and play.”* (Father_9)

### Neighbourhood issues.

Neighbourhood safety profoundly shaped parenting. In addition to financial and educational aspirations, some fathers took a protective approach by limiting their children’s exposure to negative influences. They believed that curbing their child’s exposures and their guidance were crucial in preventing their children from adopting unsuitable behaviours.

*In our childhood, we used to run around freely. But now, with many children going missing, the situation is different. Back then, people would notice and care about others’ children. If a child were missing, they would make sure to send them home. However, now, if a child is even missing for a few minutes, parents become very stressed. When we send our children out to play now, we keep a close eye on them, which was not necessary when we were children. People used to look out for each other, but now it is hard to find that sense of community, even on the next street.”* (Father_1)

*“As we have seen, children tend to become like their environment. We only send them outside when it is appropriate. Children absorb approximately 70 percent of their environment, which influences whether they develop into good or bad individuals. So, we try not to send them outside too much and make them to play at home.”* (Father_3)

### Societal expectations.

Fathers expressed varying perspectives on societal expectations and their influence on parenting. Some felt pressure to meet social standards, ensuring their children had the best education, nutrition, and opportunities to avoid criticism. Others, however, prioritized their values over societal judgment. They emphasized that parenting decisions should be guided by what is best for their children rather than external opinions.

*“We need to do all good things; otherwise people will laugh at us. Since we have all the good facilities here, we can do. We are enrolling our children in the best schools. We are providing them with nutritious food and good education, etc. We must also adapt to the circumstances of the environment in which we live. Just as a mango ripens when placed with other ripe mangoes, but won’t ripen if kept with a green one, we too must act according to our surroundings. Otherwise, people will question that despite having the means, why are we not fulfilling our responsibilities.”* (Father_2)

*“I am not bothered about what people think or say. I have experienced struggles in my life, and I will try my best to ensure that my children do not experience them. I am not going to let any other person’s opinions influence me.”* (Father_8)

C. **Parenting styles**

Fathers’ work schedules, the age of the child, and their relationship with their partner shaped parenting styles. Fathers described the time they spent with their children and how, despite limited time, they prioritized spending time with their family. Their parenting styles also adapted to their children’s age, with differences between younger children and older. Their approaches were evolving in response to their children’s changing needs, with a protective oversight that encouraged independence. They made sure to model good behaviour as a template for children to follow, rather than repeating their own parents’ caregiving practices. In some households, fathers described parenting as a shared effort, shaped by mutual respect and complementary roles in decision-making and daily care.

1. **Prioritizing quality time**

Fathers prioritized quality time despite competing demands, engaging in activities such as shared meals, recreational outings, and playing together. Many preferred staying at home rather than socializing outside.

*During school holidays, I get to spend time with him. We spend time playing games. He talks a lot and laughs so much that we enjoy it. I especially enjoy listening to the way he talks; he makes me laugh a lot.”* (Father_10)

*“I like going on walks with him. Most of the time, I just come home and spend time with my kids. I do not go out much. It is better to spend time with your children than to be outside.”* (Father_1)

*“I don’t spend much time with friends. I go out with their mother and children. My wife also likes to go out, so we all go together.”* (Father_4)

2. **Developmental awareness**

For younger children (4–6 years), fathers focused on hands-on caregiving, such as helping with homework, drop-offs, and playing with toys. However, for their older children (with some fathers having other children over six years old), they mentioned that their strategies shifted toward encouraging autonomy while maintaining discipline through authority. Fathers of older children explained how they no longer played with their children, especially with the use of smartphones among their children.

*“He does not come close to us. I would love to play with him, but he prefers to stay on his own. But he seems happy with his phone. He is growing up now, and I understand, so we do not need to be with him all the time.”* (Father_7)

*“Yes, it is necessary to give them freedom and sometimes some money also. Otherwise, they might get upset. We also need to adapt to the times. I see how things are now. So, it is important to give our children freedom.”* (Father_5)

3. **Leading by example: Modelling values through behaviour**

A foundation of fathers’ parenting was leading by example. Fathers emphasized modelling respect and the responsibility of their actions, believing that children learn more from observation than from instruction. They also modelled respectful attitudes towards their spouse to show their children about healthy relationships.

*“When there are arguments, I go outside to clear my mind. My wife might also come talk to me but outside. There is an understanding between us. I usually go out, have some tea to calm down, and then go to sleep. If I get angry, it upsets her, so I try to handle things calmly.”* (Father_5)

*“They need to learn good things from us. As parents, we are the ones they will look up to and learn from. Outside, if something bad is happening that we cannot control, we can move to a new house. However, at home, if they encounter problems, they will also learn from them. If they witness conflicts at home, they will be affected. By modelling good behaviour and discussing our issues privately, we can help them develop positive habits.”* (Father_7)

*“Humanity comes first. When I see beggars, I often buy them food. My son asks why, and I tell him that they are just like us. Sometimes, when we shop, I buy extra items. He asks why, and I explain that it is for donation. When he wonders why we should give things away, I tell him that others have children too, and not everyone is as fortunate as we are. God has given us enough, but some people struggle. After observing this a few times, he now gives to others himself.”* (Father_1)

“*If we treat them nicely, they will also behave nicely. If we shout all the time, they will grow up angry. So we have to take care of these things*.” (Father_8)

4. **Collaborative decision-making and division of labour**

In some households, fathers and mothers jointly made decisions about discipline, education, and childrearing. These fathers expressed the importance of supporting their partners and *vice versa*, believing in mutual respect and shared responsibility. They also identified their strengths and divided responsibilities, including discipline and education, among many others.

*“Both of us are responsible for education, for moral responsibilities, for their health. We take decisions together and support each other, only then our child will grow well and have a bright future. In terms of studies, wife is more dominating but regarding food and clothes I am responsible. I do not have much knowledge about studies so I left it on her.”* (Father_7)

*“It should be out moral duty to help the wives. If we distribute the responsibility, then the work can be done quickly and easily. We cannot place all the responsibility on our wives and simply relax at home. When I help my wife, she feels happy too. She has the trust that I will help her. She knows that even if I fall sick, my husband is there to take care of me and the house. Otherwise, wives are always worried about what will happen if they cannot do things, they are always worried, so that should not be there. She should also live her life without so much worry.”* (Father_2)

**Fig 1:**
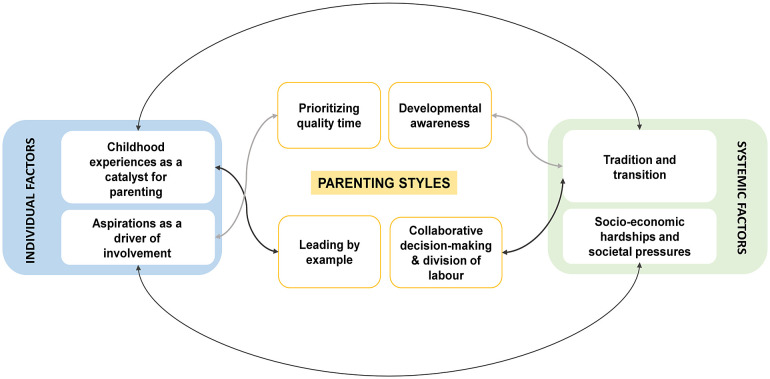
Thematic map highlighting the interaction between themes and sub-themes.

Taken together, Fig 1 highlights the interplay between the themes and sub-themes in shaping fathers’ caregiving in urban poor settings of Bangalore.

## Discussion

This study presents the lived realities of fathers in urban Bangalore, India, where fathers engage in various forms of caregiving amidst individual and systemic challenges. The study, viewed through an intersectional lens, reveals how fathers’ caregiving practices are shaped by the overlapping influences of intergenerational experiences, aspirational parenting, and constrained by structural barriers such as time poverty, gender norms, and neighbourhood instability. Despite these challenges, fathers desire or strive to remain engaged in their children’s lives. While these findings emerge from a localized context, they resonate with broader themes in global fatherhood research in comparable low-resource settings [38].

While prior research has shown fathers primarily as financial providers, fathers prioritising children’s schooling, and work-related constraints on hands-on involvement, our study situates fathers’ caregiving within the intersection of personal experiences, structural constraints, and evolving parenting. Through this exploratory study, we show how these factors combine in everyday parenting in low-resource urban settings. Fathers’ narratives reveal that caregiving is produced at the intersections of personal experiences (such as adverse childhood experiences and aspirations for their children motivate their involvement), structural constraints (such as precarious work, limited education, and neighbourhood insecurity) and generational differences and expectations. Taken together, these influences in urban poor settings show that caregiving cannot be reduced to the “provider-only” model. Rather, caregiving reflects contextually negotiated forms of masculinity with alternative practices while traditional provider ideals also co-exists. Drawing on the literature, we argue that fathers’ narratives reveal both the pressures of hegemonic norms and the emergence of alternative, caregiving-oriented masculinities that are shaped by intergenerational experiences and structural constraints [39,40]. Finally, we note that future work should test whether these negotiated caregiving patterns are associated with intra-household power dynamics and are either risk or protection with respect to gender-based violence. Many fathers described growing up in contexts marked by poverty, limited education, and emotional neglect. These early experiences were powerful motivators for what could be described as “compensatory parenting” [21,41]. Thus, they made deliberate efforts at actively engaging with their children to counteract the “provider-only” role they had witnessed in their own upbringing. Fathers expressed the need to change with the times, and their conscious rejection of harsh discipline in favour of listening, playing, and encouragement. However, for others, these hardships persisted into their own fatherhood as well, thus constraining their ability to be more involved despite their wants and intentions. This intergenerational shift is similar to findings from across the world, where adverse childhood experiences motivated some parents to nurture despite scarce resources [42,43], though ongoing hardships still limit the ability of those who continue to face them. Education emerged as a core aspiration, with fathers linking their own limitations in educational attainments to limited life chances and thus were determined to secure better educational opportunities for their children [44,45]. However, due to their inconsistent work hours and informal labour which offer few protections, some engaged in symbolic caregiving including buying gifts or school supplies, planning weekend outings, etc. These acts were a show of their commitment as fathers who were genuinely invested caregivers. Such strategies align with research showing that material gifts [46,47] and structured “father–child activities” [43,48] can foster emotional bonds even when time is constrained.

Our study also noted the presence of rigid gender norms that continued to define caregiving as a woman’s domain, even though some fathers reflected more gender-equitable role and ideals. Most fathers believed “our responsibility is to earn” with caregiving seen as the mother’s responsibility – often by necessity due to economic instability as observed in the MAASTHI cohort, mothers typically assumed the bulk of domestic and childcare duties [31]. Yet some fathers described co-parenting in terms of stepping in when their wives were ill, making schooling decisions, or jointly disciplining their children. These subtle shifts in fathers’ involvement are noteworthy. However, we must also note that these fathers also referred to some of these actions as “helping” their wives, demonstrating how gender and their roles continue to shape their perception and willingness to engage in domestic labour [49,50].

Furthermore, systemic inequities, including financial constraints, poor neighbourhood safety, and gendered societal expectations, limit parenting capacities. For most fathers living in urban poor settings as ours, the unpredictable incomes from informal work meant that even missing a single day of work could mean no food on the table, thus spending time with children is a luxury few could afford. Compounding this, neighbourhood instability with substance misuse, poor housing, unsafe roads, etc. forced hyper-vigilant parenting that affected children’s autonomy as well as peer interaction. Fathers in our study reflected on societal expectations around caregiving, where some felt compelled to conform, while others expressed indifference. However, these expectations often circled back to gendered caregiving roles, with mothers continuing to bear the primary responsibility. This dynamic can leave fathers feeling excluded or uncertain and unclear about their parenting roles. The persistent breadwinner-homemaker divide, coupled with maternal gatekeeping behaviours, may limit fathers’ opportunities for involvement [51]. Additionally, weak labour laws, such as the absence of paternity leave, lack of job security, and poor occupational health safeguards, particularly for informal workers, add to the challenges faced by fathers. These systemic constraints, along with workplace cultures that discourage paternal caregiving can lead to stress, emotional isolation, eventually impacting fathers’ mental well-being and their engagement in child-rearing [38,52]. Despite facing economic constraints in urban poor settings, fathers in this study demonstrated a deep commitment to providing for their families, balancing financial responsibilities with interest in active parenting, presenting a nuanced understanding of their roles as both providers and caregivers.

Parenting styles kept evolving and adapting based on their experiences and situations. As children aged, fathers reported evolving their roles from direct care to granting independence. Several fathers described shifting from hands-on play in early years to supervising homework and understanding the need for autonomy for their children. However, the incessant use of smartphones by their children created new tensions with fathers bothered about screen time, as they expressed concern about screen time and a loss of face-to-face interaction, reflecting anxieties around digital advances and intergenerational divides [53,54]. These changes show the dynamic nature of fatherhood shaped by several factors present in urban poor settings.

Hence, we note that fathers’ caregiving practices in low-income urban settings are shaped by a complex interplay between individual experiences, systemic structures, and their own evolving parenting styles. Many fathers drew motivation from their childhoods and a desire to break cycles of hardship, which influenced their aspirations and involvement in their children’s lives. However, these intentions were also constrained by broader systemic factors such as financial insecurity, rigid gender norms, and unsafe neighbourhood environments. As a result, fathers adapted their parenting styles with prioritising quality time spent with children at home, adjusting their involvement based on their child’s age and developmental needs, and trying to be more present than their own fathers. This interconnectedness suggests that fathering cannot be understood in isolation; rather, it is shaped by the dynamic interaction between individual experiences, structural barriers, and day-to-day interactions with their family, community, and cultural context.

We also consider our findings from this exploratory study alongside complementary evidence from interviews with mothers and grandmothers in the same MAASTHI cohort [55]. The previously published study noted that mothers and grandmothers engage in caregiving while dealing with competing household demands, and households with absent fathers, increases both caregiving burdens and social stigma on single mothers. Taken together, these perspectives provide a fuller picture of household caregiving in these low-resource urban settings. Fathers’ accounts of wanting to break cycles of hardship and prioritising children’s education must be noted against mothers’ and grandmothers’ descriptions of everyday caregiving, the role of kin networks.

Comparative evidence from African settings also shows how deeply entrenched gendered caregiving roles can be reshaped through targeted interventions. Community-led parenting programmes in Kenya and Zambia, for instance, have led to increased father involvement in childcare and household work, suggesting that structural change is possible when supported by inclusive programming. Moreover, research shows that increased father involvement in caregiving has also been associated with reduced maternal stress and improved maternal mental health [52,56]. Such engagement not only alleviates the caregiving burden on mothers but also enhances family well-being and child development outcomes, which aligns with Sustainable Development Goal 5 of the United Nations to achieve gender equality and empower all women and girls [57].

To address the identified gaps, it is essential to integrate fathers into early childhood development interventions moving beyond mother-centric approaches. Evidence shows that father-inclusive programmes can improve child development outcomes and reduce caregiving burdens on women [38]. Further, Nair *et al* through their intervention in India also showed that fathers benefit from community‑and technology‑driven support to build their caregiving skills and confidence [58].Community-level strategies such as father peer groups, gender-equity media messages can challenge patriarchal norms and promote collaborative caregiving. Further, systemic inequities can be addressed by promoting workplace reforms, expanding paternity leave, and investing in safe neighbourhood infrastructure. To advance caregiving equity, policies must also offer tailored resources that that enhance fathers’ confidence, build their caregiving skills, and support them to manage their own well-being thus enabling them to play a meaningful role in their children’s growth and development. By recognizing fathers as co-caregivers, not just economic providers, such efforts can advance caregiving equity in the Global South. Hence, there is a pressing need to amplify the voices of fathers from urban poor areas of the Global South in policy discourse to ensure that interventions reflect grassroots realities.

We frame these findings through an intersectionality lens to emphasise that gender interacts with other social positions and contexts in shaping caregiving. In our analysis we focused on gender and socio-economic position, and also examined how the child’s age or difference in generation, employment type, educational attainment, appear together in fathers’ narratives. Using matrix coding and code co-occurrence we identified recurring patterns where two or more of these axes jointly shaped caregiving choices (for example, childhood adversity and precarious work could influence protective parenting and/or aspirational caregiving). Since this was an exploratory father-centred study we therefore describe observed qualitative intersections and recommend future dyadic and larger studies to test intersections more systematically.

### Strengths and limitations

This study’s key strength lies in bringing forth the voices of urban poor fathers from South India – a population rarely represented in early childhood or gender equity research. The inclusion of fathers from diverse age groups, occupations, educational backgrounds, and family structures ensured that our findings reflected real-world variations in parenting. While qualitative research is not dependent on numerical adequacy as in quantitative studies, after our study completed ten in-depth interviews, and mindful of the exploratory nature of this study and the resource limitations, we noted that the data showed recurring information with no emergence of new insights. Therefore, we concluded data collection after interview 10, and believe that although our findings are not statistically generalizable, the study provides contextual insights that may be transferable to similar urban poor settings in the Global South. We also note that social desirability bias may have influenced participants’ responses, with fathers potentially presenting idealized caregiving practices to align with perceived societal norms or to make a favourable impression on the interviewer. Due to the exploratory nature of the study, another limitation is that mothers were not interviewed hence factors such as maternal gatekeeping, or differences in reporting between parents may therefore help explain why fathers framed tasks as “helping” rather than shared caregiving. Hence, this presents an opportunity for future research to build on this exploratory work by employing larger, more geographically diverse samples and by including mothers alongside fathers Such studies would deepen understanding of evolving gender roles in caregiving and to gather their perspectives regarding the role of fathers in caregiving.

## Conclusion

Narratives of fathers in Bangalore’s urban poor communities challenge reductive stereotypes, showing their intentional strategies to disrupt cycles of disadvantage through education, emotional bonds, and adaptive parenting. While aspirations drive involvement, structural inequities continue to perpetuate efforts of involved parenting. By contextualizing paternal caregiving within the lived realities of urban fathers in South India, this exploratory study contributes to the broader discourse on inclusive caregiving policies and fatherhood in the Global South. These challenges can help inform policies and interventions aimed at fostering more equitable and supportive caregiving environments. Our findings call for targeted policies that integrate fathers into early childhood programmes in disadvantaged urban settings by addressing challenges through tailored interventions and resources.

## Supporting information

S1 File Consolidated Criteria for Reporting Qualitative Research (COREQ) checklist. (PDF)

S2 File Coding matrix of the in-depth interviews. (PDF)
