# Supplementary material for: Becoming fathers, becoming caregivers: A qualitative exploration of intersectional influences shaping caregiving in an urban poor South Indian setting
Source: PLoS One. 2025 Oct 23;20(10):e0334717. doi: 10.1371/journal.pone.0334717 (PMC12548844; doi:10.1371/journal.pone.0334717)
Supplement: S2 File — (PDF) [file pone.0334717.s002.pdf]

Theme	Sub-theme	Codes	Father _1	Father _2	Father _3	Father _4	Father _5	Father _6	Father _7	Father _8	Father _9	Father _10
Individual factors	Childhood experiences as a catalyst for parenting	childhood struggles & responsibilities	Yes	No	Yes	Yes	Yes	No	No	No	No	Yes
		limited caregiver involvement	Yes	No	Yes	No	Yes	No	No	Yes	No	Yes
		mitigating childhood experiences	Yes	Yes	Yes	Yes	Yes	Yes	Yes	Yes	Yes	Yes
	Aspirations as a driver of involvement	emotional fulfilment through parenting	No	No	No	No	No	Yes	No	Yes	Yes	No
		Parents aspirations	Yes	Yes	Yes	Yes	Yes	Yes	No	Yes	No	Yes
		limited education	No	No	Yes	No	Yes	No	No	No	No	No
Systemic factors	Tradition and transition	difference in parenting style from one's own parents	Yes	Yes	Yes	Yes	Yes	Yes	Yes	No	Yes	Yes
		learning parenting	Yes	No	No	Yes	No	No	No	No	No	No
		parenting according to locality or place of stay	Yes	Yes	No	No	No	No	No	No	No	No
		influence of parents upbringing	No	Yes	No	Yes	Yes	No	Yes	No	Yes	Yes
		constantly with mother	No	No	No	No	No	Yes	No	Yes	No	No
		father is sole provider of finances	Yes	No	Yes	No	No	Yes	No	Yes	No	Yes
		mother's responsibilities	Yes	Yes	Yes	No	Yes	Yes	Yes	Yes	Yes	No

Theme	Sub-theme	Codes	Father _1	Father _2	Father _3	Father _4	Father _5	Father _6	Father _7	Father _8	Father _9	Father _10
	Socio-economic hardships and societal pressures	neighbourhood effect	Yes	Yes	Yes	Yes	Yes	Yes	No	Yes	Yes	Yes
		income issues	No	No	No	No	Yes	No	No	No	No	No
		societal views	Yes	Yes	Yes	Yes	Yes	No	No	Yes	No	No
		safety concerns	Yes	Yes	Yes	Yes	No	No	No	Yes	No	Yes
		unsafe community	Yes	No	Yes	Yes	Yes	No	No	Yes	Yes	No
		protective parenting	Yes	Yes	Yes	Yes	Yes	Yes	No	Yes	Yes	Yes
		less time due to work	Yes	Yes	Yes	No	Yes	Yes	Yes	Yes	Yes	Yes
Parenting styles	Prioritizing quality time	caregiver led activities	No	Yes	No	No	No	No	No	No	No	No
		importance of spending time	No	Yes	Yes	No	No	No	No	No	No	No
		spending more time with family rather than socializing	Yes	Yes	Yes	No	No	No	No	Yes	Yes	No
	Developmental awareness	parenting strategies	Yes	Yes	Yes	Yes	Yes	Yes	Yes	Yes	No	Yes
		changing parenting styles with time	No	Yes	Yes	No	Yes	No	Yes	Yes	No	No

Theme	Sub-theme	Codes	Father _1	Father _2	Father _3	Father _4	Father _5	Father _6	Father _7	Father _8	Father _9	Father _10
	Leading by example	what parents deem absolutely necessary	Yes	Yes	Yes	Yes	Yes	Yes	No	Yes	No	Yes
		modelling positive behaviour	No	Yes	No	No	Yes	No	No	Yes	No	Yes
		moral and cultural values	No	Yes	Yes	Yes	Yes	No	Yes	Yes	No	Yes
		coping with marital issues	No	No	Yes	No	Yes	No	No	Yes	Yes	Yes
	Collaborative decision-making and division of labour	dealing with child's behaviour/ disciplining	Yes	Yes	Yes	Yes	No	Yes	Yes	Yes	Yes	Yes
		shared responsibilities	Yes	Yes	Yes	Yes	No	Yes	No	Yes	Yes	Yes
